# Effect of MT3 on Retinal and Choroidal TGF-*β*2 and HAS2 Expressions in Form Deprivation Myopia of Guinea Pig

**DOI:** 10.1155/2017/5028019

**Published:** 2017-10-15

**Authors:** Tao Li, Xiaodong Zhou, Bing Li, Bo Jiang

**Affiliations:** ^1^Department of Ophthalmology, Jinshan Hospital of Fudan University, Shanghai, China; ^2^Department of Central Laboratory, Jinshan Hospital of Fudan University, Shanghai, China

## Abstract

**Purpose:**

To confirm its dose-dependent effect on form deprivation myopia and evaluate the effect of MT3 at different tissue concentrations on changes in mRNA and protein expression for TGF-*β*2 and HAS2.

**Methods:**

MT3 was intravitreally injected into deprived eyes at two-day intervals. Refraction was measured by streak retinoscopy after cycloplegia. The axial dimensions were measured by A-scan ultrasound. The quantitative RT-PCR and Western blot were used to detect the changes of TGF-*β*2 and HAS2 expressions in the retina and choroid of guinea pigs.

**Results:**

MT3 treatment produced a significant dose-dependent reduction in relative myopia compared to FD group (both *p* < 0.001). There were statistically significant increases in retinal and choroidal mRNA levels for both TGF-*β*2 and HAS2 after injections of 10 *μ*M of MT3, when compared to the FD group. There were no significant differences in retinal and choroidal TGF-*β*2 protein expression levels between the MT3 treatment groups and FD group (all *p* > 0.05). The injections of 10 *μ*M of MT3 caused a marked decrease in retinal HAS2 protein expression level, when compared to the FD group (*p* = 0.001).

**Conclusion:**

MT3 can inhibit form deprivation myopia, and MT3 treatment can result in changes of retinal and choroidal TGF-*β*2 and HAS2 mRNA and protein expressions.

## 1. Introduction

Myopia is an increasingly widespread condition around the world, particularly in Eastern Asia [1–3]. Excessive axial elongation is responsible for the development of myopia. High myopia is characterized by a progressive elongation of the eye ball and scleral thinning. Those with high myopia are at a high risk of subsequent retinochoroidal pathologies and blindness [4, 5]. This emphasizes the importance of controlling the myopic development by slowing axial elongation.

A nonselective muscarinic antagonist atropine has been shown to inhibit myopia progression in children; however, atropine has the deleterious side effects of cycloplegia and photophobia [6]. A partially selective muscarinic antagonist pirenzepine had been in clinical trials for several years with milder side effects, before the study was abruptly terminated [7–9]. Studies on several animal models found that pirenzepine was effective at reducing form deprivation myopia (FDM) in a dose-dependent manner [10]. The selectivity for M1 receptors of pirenzepine is only fourfold higher than that for M4 receptors [11], so it is clear that pirenzepine is also binding to M4 receptors. These findings highlight the problem of identifying which specific muscarinic receptors are responsible for myopia inhibition. Furthermore, M4 receptor is expressed in the retina and choroid in guinea pigs [12]. MT3, a selective M4 muscarinic receptor antagonist, has been demonstrated to have 102-fold higher selectivity for the M4 receptor than for the M1 receptor [13]. It is reported that MT3 effectively inhibits the development of FDM in animal models [14–16].

An important goal of research into experimental myopia has been to identify the components of the retinal and choroidal signalling cascades responsible for transduction of the blur signal and transmission of this information from these tissues to the sclera. Transforming growth factor (TGF)-*β* is a multifunctional cytokine, with TGF-*β*2 being the predominant isoform in the ocular tissues [17]. The reports of TGF-*β*2 expression in experimental myopia were controversial. Seko et al. [18] reported that the TGF-*β*2 content was increased in the retina-RPE-choroid and sclera in myopic chick eyes by ELISA. Kusakari et al. [19] also verified this change of TGF-*β*2 by immunohistochemistry. But Honda et al. [20] found that both protein and mRNA levels of TGF-*β*2 were reduced in the retina-RPE-choroid in myopic chick eyes. In a tree shrew model of myopia, Jobling and colleagues found that mRNA expressions of TGF-*β*2 isoforms were reduced in the sclera [21, 22], but the protein and mRNA expressions of TGF-*β*2 isoforms in the retina were not significantly changed [23]. Hyaluronan (HA) was a nonsulfated glycosaminoglycan distributed widely throughout connective, epithelial, and neural tissues [24]. Hyaluronan synthase 2 (HAS2) was responsible for the synthesis of HA [24]. HAS2 gene expression of the choroid in form-deprived chick eyes was not significantly different from controls, but increased significantly during recovery period [25]. The accumulation of HA within the choroidal stroma likely accounted for the rapid thickening of the choroid during compensation for myopic defocus. The rapid decrease in HA levels was observed in the sclera during negative lens compensation [26]. But, little is known about the HAS2 changes of retina in experimental myopic eyes.

We explored the effects of MT3 on FDM in the guinea pig and the effects of MT3 treatment on mRNA and protein expressions for TGF-*β*2 and HAS2 in the retina and choroid in the guinea pig. In a first step, we confirmed its dose-dependent effect on form deprivation myopia in the guinea pigs. Since TGF-*β*2 and HAS2 may be involved in the development of form deprivation myopia [18–20, 25], in a second step, we studied the effect of MT3 at different tissue concentrations on changes in mRNA and protein expression for TGF-*β*2 and HAS2.

## 2. Methods

### 2.1. Animals

Three-week-old pigmented guinea pigs were obtained from the breeding room in Thai town, Fengxian District, Shanghai City, China. These guinea pigs were raised under a 12–12 h light-dark cycle. The room temperature was maintained at 25°C. The animal research was approved by the Animal Care and Ethics Committee at Jinshan Hospital of Fudan University, Shanghai, China. The treatment and care of the animals were consistent with the ARVO Statement for the Use of Animals in Ophthalmic and Vision research.

Animals were allocated into one of four experimental groups. The form deprivation (FD) group consisted of animals that underwent no intravitreal injection, just FD (*n* = 8). The FD + 2.5 *μ*M MT3 group and FD + 10 *μ*M MT3 group consisted of animals that received a 10 *μ*L injection of MT3 (Peptides International, http://pepnet.com/) at a concentration of either 2.5 *μ*M (*n* = 16) or 10 *μ*M (*n* = 16) and FD. These doses were chosen on the basis of a previous work which showed them to produce suppression of the excessive axial elongation associated with FD in chicks [14]. The control group consisted of animals without injection and FD (*n* = 8).

### 2.2. Form Deprivation Myopia

The procedure of form deprivation has been detailed in the previous studies [27, 28]. Briefly, the right eyes of guinea pigs were covered with face masks. These face masks were made by latices, which were opaque and soft with light transmission of 60%. The face masks did not contact with the cornea, which made the right eyes blink freely. To ensure the face masks are in place, a routine check-up was made once a day.

### 2.3. Treatment Protocols

Guinea pigs were intraperitoneally injected with 100 mg/kg ketamine HCL (Gutian Pharmaceutical Company, Fujian, China). After anesthesia, the right eyes of the guinea pigs received 7 intravitreal injections through the pars plana 1 mm behind the limbus using a 26-gauge needle at two-day intervals in 2 weeks according to their treatment group. After the last intravitreal injection, the right eyes of the guinea pigs continued to be occluded for another 2 weeks. The total treatment periods were 4 weeks. The face masks were renewed after every day.

### 2.4. Refraction and Biometric Measurement

The manipulation of refraction and biometric measurements has been detailed in a previous study [28]. Each eye was measured before treatment and after 4 weeks of treatment. One drop of tropicamide 0.5% was topically administered to the eye every 5 minutes for four times to achieve cycloplegia and a completely dilated pupil. Ocular refraction was measured with a streak retinoscope 30 minutes after the last drop. Refractions were reported as spherical equivalents (SE). SE = spherical degree + 0.5 × cylindrical degree.

The axial dimensions were measured by A-scan ultrasound (11 MHz; Hiscan A/B, Opticon, Italy), which consisted the axial length and vitreous length. Topical corneal anesthesia was administered with oxybuprocaine hydrochloride 0.4% (Santen, Japan) before the ultrasound measurement. The ultrasound probe had direct contact with the cornea during the axial measurement. A genuine measurement was confirmed when clear traces of various components of the eye with consistent waves and amplitudes were detected [29]. The average value of the 10 repeated measurements was then analyzed. All measurements were performed by the same examiner who was masked to the treatment group assignment.

### 2.5. Tissue Isolation

After a 4 week treatment period, animals were terminally anesthetized and the right eyes in all animals were enucleated. The enucleated eyes were hemisected. The posterior retina and choroid were isolated by a 7 mm surgical trephine. The neural retina and choroid was then carefully removed from the retinal pigment epithelium and underlying sclera. Tissues were stored in liquid nitrogen at the temperature of −80°C until use.

### 2.6. Real-Time PCR (RT-PCR)

Total RNA from the retina or choroid tissue in a form-deprived eye was isolated using TRIzol (Invitrogen, Carlsbad, CA) according to the manufacturer's protocol. The quantitative RT-PCR analysis was performed using the two-step RT-PCR kit with SYBR Green (Takara, Shiga, Japan) on a Thermal Cycler Dice TP800 sequence detection system (Takara, Shiga, Japan), according to the manufacturer's instructions. At the end of the amplification, cycle threshold (Ct) values of each reaction were acquired using the SDS v1.4 software. Then, the relative quantification of target gene expression was calculated by the 2^−ΔΔCt^ method. The sequences of used primer pairs and length of the amplified sequences were shown in Table 1. GAPDH served as a control.

### 2.7. Western Blot

Retina and sclera tissues were homogenized, respectively, and 5 *μ*g of homogenate was loaded for SDS-PAGE. Proteins were separated on a 10% resolving gel and transferred to poly cellulose acetate membrane (Millipore).Then these membranes were blocked with 5% nonfat dry milk in 0.1% Tween 20 (TBS-T; 2 mmol/L Tris-HCl, 50 mmol/L NaCl, pH 7.4) for 2 hours at a room temperature. Afterwards, these membranes were then cultured overnight with a primary antibody against TGF-*β*, HAS, or GAPDH at a 1 : 1000 dilution at the temperature of 4°C in the blocked buffer. Furthermore, these membranes were washed with 0.1% Tween 20 and then treated with goat anti-rabbit IgG conjugated to alkaline phosphatase (1 : 5000) for 1 hour at the temperature of 37°C. Stripping filters and reprobing for GAPDH were normalized. The controls of nonspecific binding were determined by the absence of primary antibodies. A film scanner (Image Master VDS; Amersham Biosciences Inc., Piscataway, NJ) was used to scan the films.

### 2.8. Statistical Analysis

All data were expressed as the mean ± SD and were analyzed using SPSS version 17.0 software (SPSS Inc., Chicago, IL, USA). Statistical comparisons between groups were made using one-way analysis of variance (ANOVA) with the Bonferroni post hoc test. *P* < 0.05 was considered statistically significant.

## 3. Results

### 3.1. Effect of MT3 on Myopia Development in Guinea Pigs

**Figure 1:**
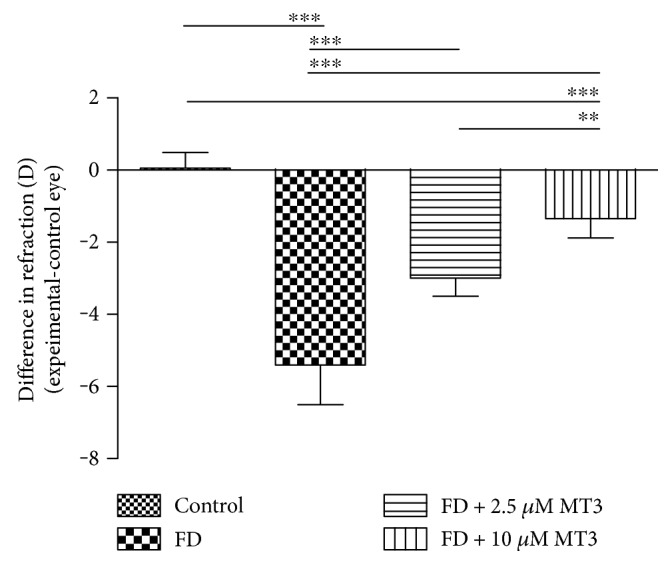
Differences in ocular refraction measures between the right and left eyes for the control, FD, FD + 2.5 *μ*M MT3, and FD + 10 *μ*M MT3 groups. FD: form deprivation. ^∗∗^*p* < 0.01 and ^∗∗∗^*p* < 0.001.

**Figure 2:**
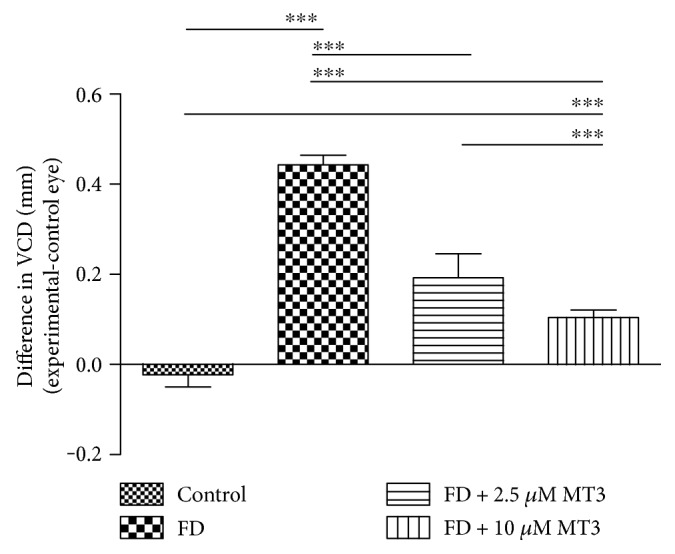
Differences in VCD measures between the right and left eyes for the control, FD, FD + 2.5 *μ*M MT3, and FD + 10 *μ*M MT3 groups. VCD: vitreous chamber depth; FD: form deprivation. ^∗∗∗^*p* < 0.001.

**Figure 3:**
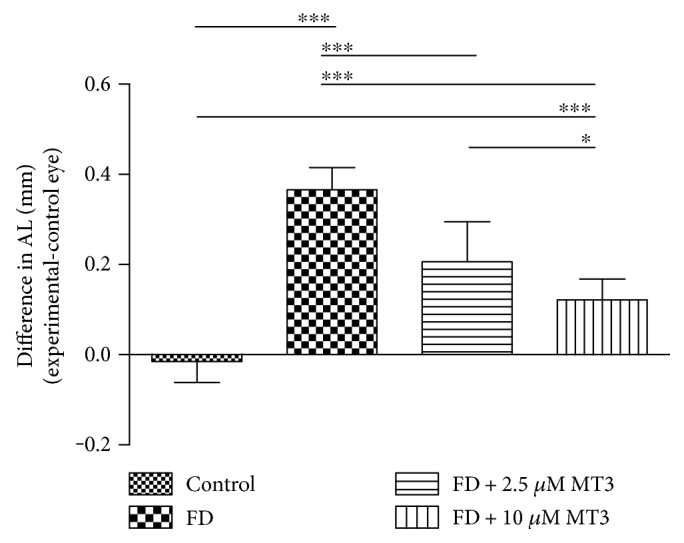
Differences in AL measures between the right and left eyes for the control, FD, FD + 2.5 *μ*M MT3, and FD + 10 *μ*M MT3 groups. AL: axial length; FD: form deprivation. ^∗^*p* < 0.05 and ^∗∗∗^*p* < 0.001.

Both the 2.5 *μ*M and 10 *μ*M doses of MT3 reduced the amount of relative myopia (right–left eyes), compared to the FD group (Figure 1; FD −5.40 ± 1.10 D, 2.5 *μ*M −3.00 ± 0.50 D, 10 *μ*M −1.35 ± 0.53 D), with both doses reaching significance (*p* < 0.001 for both). The percentage reductions in myopia due to MT3 were 44% (2.5 *μ*M) and 75% (10 *μ*M). Compared to the FD group, both doses of MT3 also reduced the relative vitreous chamber depth (VCD) between the right and left eyes (Figure 2; FD 0.45 ± 0.02 mm, 2.5 *μ*M 0.19 ± 0.05 mm, and 10 *μ*M 0.10 ± 0.02 mm; *p* < 0.001 for both) and axial length (AL) differences (Figure 3; FD 0.37 ± 0.05 mm, 2.5 *μ*M 0.21 ± 0.09 mm, 10 *μ*M 0.12 ± 0.05 mm; *p* < 0.001 for both). The relative myopia, VCD, and AL differences in the FD + 10 *μ*M MT3 group were smaller than those in the FD + 2.5 *μ*M MT3 group (*p* = 0.001 for relative myopia, *p* < 0.001 for VCD, and *p* = 0.022 for AL, resp.), but larger than those in the control group (all *p* < 0.001).

### 3.2. Effect of MT3 on Retinal and Choroidal TGF-*β*2 and HAS2 mRNA Expression

**Figure 4:**
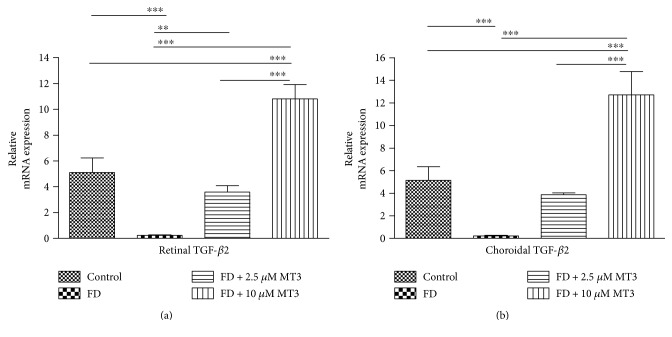
TGF-*β*2 relative mRNA expression for retinal (a) and choroidal (b) samples was calculated using the quantitative RT-PCR relative to GAPDH. ^∗∗^*p* < 0.01 and ^∗∗∗^*p* < 0.001.

**Figure 5:**
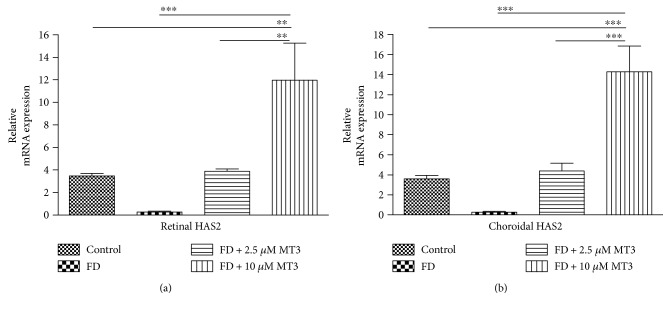
HAS2 relative mRNA expression for retinal (a) and choroidal (b) samples was calculated using the quantitative RT-PCR relative to GAPDH. ^∗∗^*p* < 0.01 and ^∗∗∗^*p* < 0.001.

FD caused significant retinal and choroidal TGF-*β*2 mRNA level decreases when compared to control eyes (both *p* < 0.001), but did not caused significant retinal and choroidal HAS2 mRNA level changes (both *p* > 0.05; Figures 4 and 5). As shown in Figures 4 and 5, the retinal and choroidal TGF-*β*2 and HAS2 mRNA with injection of 10 *μ*M of MT3 were higher than the FD group (all *p* < 0.001), control group (*p* < 0.001 for TGF-*β*2 in retina, *p* < 0.001 for TGF-*β*2 in choroid, *p* = 0.001 for HAS2 in retina, and *p* < 0.001 for HAS2 in choroid, resp.), and FD+ 2.5 *μ*M MT3 group (*p* < 0.001 for TGF-*β*2 in retina, *p* < 0.001 for TGF-*β*2 in choroid, *p* = 0.002 for HAS2 in retina, and *p* < 0.001 for HAS2 in choroid, resp.). The retinal TGF-*β*2 mRNA with injection of 2.5 *μ*M of MT3 was higher than the FD group (*p* = 0.005), whereas there were no significant differences in the choroidal TGF-*β*2 mRNA and retinal and choroidal HAS2 mRNA between the FD + 2.5 *μ*M MT3 group and FD group (all *p* > 0.05). There were no significant differences in the retinal and choroidal TGF-*β*2 and HAS2 mRNA between the FD + 2.5 *μ*M MT3 group and control group (all *p* > 0.05).

### 3.3. Effect of MT3 on Retinal and Choroidal TGF-*β*2 and HAS2 Protein Expressions

**Figure 6:**
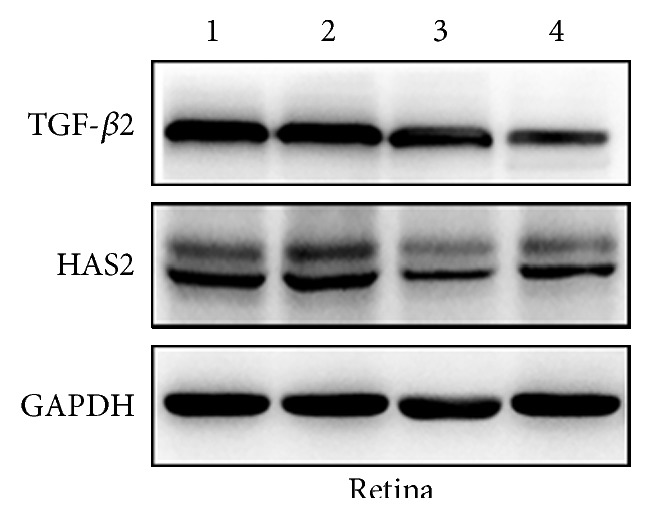
Western blot analysis of TGF-*β*2 and HAS2 expressions in the guinea pig retina. 1: control group. 2: FD group. 3: FD + 2.5 *μ*M MT3 group. 4: FD + 10 *μ*M MT3 group.

**Figure 7:**
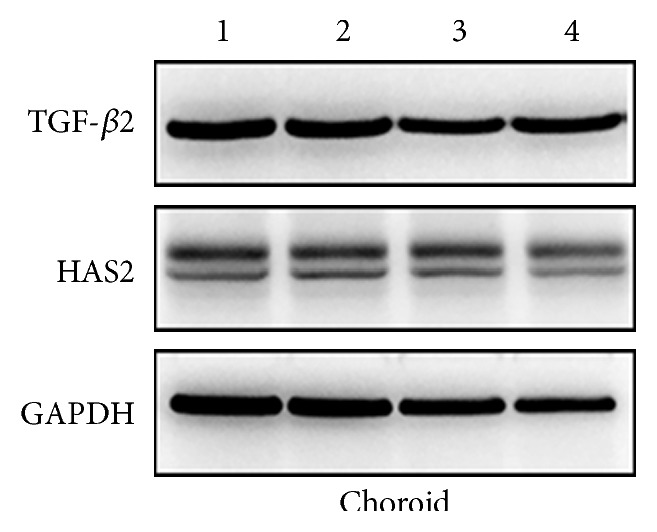
Western blot analysis of TGF-*β*2 and HAS2 expression in the guinea pig choroid. 1: control group. 2: FD group. 3: FD + 2.5 *μ*M MT3 group. 4: FD + 10 *μ*M MT3 group.

**Figure 8:**
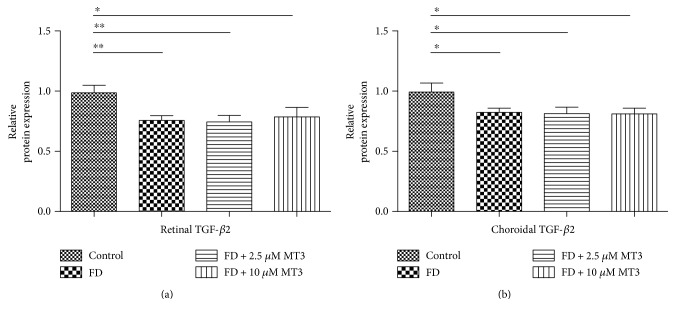
Western blot analysis of TGF-*β*2 protein expression level in the guinea pig retina (a) and choroid (b) analyzed by measuring band density and normalized to GAPDH. Protein expression levels were expressed as fold change. Data shown are mean ± SD. ^∗^*p* < 0.05 and ^∗∗^*p* < 0.01.

TGF-*β*2 and HAS2 protein expressions were detected by Western blot analysis of the retina and choroid (Figures 6 and 7). FD caused marked decrease in retinal and choroidal TGF-*β*2 protein expression levels, compared to the control group (Figures 8(a) and 8(b); *p* = 0.009 and *p* = 0.032). Similar trends of retinal (*p* = 0.007 for FD + 2.5 *μ*M MT3 group and *p* = 0.021 for FD + 10 *μ*M MT3 group) and choroidal (*p* = 0.024 for FD + 2.5 *μ*M MT3 group and *p* = 0.021 for FD + 10 *μ*M MT3 group) TGF-*β*2 protein expression levels were found in the MT3 treatment groups, compared to the control group (Figures 8(a) and 8(b)). There were no significant differences in retinal and choroidal TGF-*β*2 protein expression levels between MT3 treatment groups and FD group (all *p* > 0.05).

**Figure 9:**
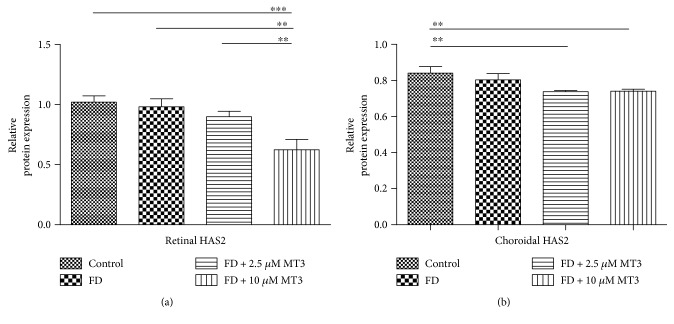
Western blot analysis of HAS2 protein expression level in the guinea pig retina (a) and choroid (b) analyzed by measuring band density and normalized to GAPDH. Protein expression levels were expressed as fold change. Data shown are mean ± SD. ^∗∗^*p* < 0.01 and ^∗∗∗^*p* < 0.001.

As shown in Figure 9(a), the injections of 10 *μ*M of MT3 caused a marked decrease in retinal HAS2 protein expression levels, when compared to the FD group (*p* = 0.001), control group (*p* < 0.001), and FD + 2.5 *μ*M MT3 group (*p* = 0.005). Furthermore, the injections of 2.5 *μ*M and 10 *μ*M of MT3 caused marked decrease in choroidal HAS2 protein expression levels, when compared to the control group (Figure 9(b); *p* = 0.007 and *p* = 0.008, resp.). There were no significant differences in retinal and choroidal HAS2 protein expression levels between the control group and FD group (both *p* > 0.05).

## 4. Discussion

The present study demonstrates that the selective M4 muscarinic receptor antagonist MT3 is effective in significantly inhibiting FDM in the guinea pigs in a dose-dependent manner. The structural cause of the reduction in myopia is predominantly inhibition of vitreous chamber elongation. MT3 treatment results in an upregulation of retinal and choroidal mRNA levels of TGF-*β*2 and HAS2; protein expression levels, however, are downregulated. Such changes may be a potential mechanism of inhibiting form deprivation myopia in the guinea pigs.

While previous studies using either broad-band muscarinic antagonists (e.g., atropine) or partially selective antagonists (e.g., pirenzepine) have demonstrated efficacy in reducing myopia, these studies have needed to use doses substantially higher than what would be considered necessary for a muscarinic receptor-based mechanism. The significance of this study was that MT3 was highly selective for the M4 receptors. Thus, the doses used in the present study were calculated to be at micromolar concentrations at the receptor, in keeping with a direct muscarinic receptor-based mechanism of action [12]. M4 receptor mechanism in myopia prevention was conserved across species (e.g., chickens and mammals). McBrien et al. [14] reported that MT3 effectively inhibited the development of FDM in chickens and prevented the choroidal thinning that was concomitant with the development of myopia. The maximal inhibition efficacy was produced by 10 *μ*M, and the minimum inhibition efficacy was produced by 2.5 *μ*M. Arumugam and McBrien [15] also verified inhibition of MT3 on FDM in tree shrew. Nickla et al. [16] found MT3 inhibited the myopia in response to form deprivation, but did not affect the compensation to negative lenses, without choroidal thinning in either condition. Data from the current study showed that a 75% inhibition of FD myopia in guinea pigs was achieved by MT3 with a presumed concentration of 10 *μ*M, whereas a 44% inhibition of myopia by 2.5 *μ*M of MT3. This indicates that MT3 is also effective in inhibiting myopia development of guinea pigs in a dose-dependent manner. But the mechanism is still unclear.

TGF-*β* plays an important role in the development of experimental myopia. Over 95% of the total TGF-*β* content in the guinea pig is TGF-*β*2 [30]. For that reason, proper characterisation of tissue-specific isoform expression in a well-defined mammalian model for myopia is important in elucidating TGF-*β*2's role in the ocular growth regulatory system. In the present study, FD caused significant decreases of retinal and choroidal TGF-*β*2 mRNA and protein expression levels compared to the control eyes in guinea pigs, which was similar to the finding of Honda et al. [20]. However, other studies reported that TGF-*β*2 content was increased in the retina-RPE-choroid or was not significantly changed [18, 19, 23]. Our results are different from the previous studies, which are complicated by several confounding factors. Firstly, mammalian experimental animals are more similar to humans. Therefore, preparing an appropriate animal model and ocular tissues is important for studies on the pathogenic mechanism of myopia. In the current study, guinea pigs were used to construct the FDM model, which were different from chickens. This discrepancy may be a result in the species-dependent regulatory roles of TGF-*β*2 in the eye growth. Chen et al. [31] found that the TGF-*β*2 expression of the sclera in the experimentally induced myopia group was significantly higher compared with the normal control group. Differences between our results and the Chen study [31] could be due to the different experimental paradigms (lens-induced myopia VS FDM). Additionally, tissue difference may be another important reason. The TGF-*β*2 expression of the retina and choroid were tested in the present study, whereas that of the sclera was tested by Chen et al. [31].

In the present study, there were no significant differences in retinal and choroidal HAS2 mRNA and protein expression levels between the FD group and control group. Rada et al. [25] found that no significant differences were detected in HAS2 mRNA in choroids of form-deprived eyes compared with the controls in chicks after 10 days of visual deprivation, but HAS2 expression significantly elevated in form-deprived eyes during recovery period. Moring et al. [26] found that the relative HA content in sclera was significantly lower in treated eyes than in control eyes in tree shrew after 1 day of negative lens wear. Then, they found that the difference in the treated eyes compared with the control eyes for HA was not significant after 2 days of treatment. HAS2 is the isoenzyme that most likely modulates HA synthesis rates [32], and decreased HAS2 mRNA levels are correlated with decreased HA biosynthesis [33, 34]. It is possible for HAS2 to be rapidly decreased and HA to be rapidly degraded in treated eyes during negative lens compensation. It is suggest that HAS2 and HA levels may play a previously unrecognized early role in regulating eyeball development.

However, levels of retinal and choroidal TGF-*β*2 and HAS2 mRNA were increased in the form-deprived eyes by MT3, with downregulations of their protein expression levels. The levels of TGF-*β*2 in the retina and choroid reflected its role in maintaining the structural and functional characteristics of the eye [35]. Thus, the data from our study suggest that TGF-*β*2 and HAS2 may be involved in retinal and choroidal regulation during myopia development. Partial inhibition of FDM with MT3 indicates that the regulation of eye growth via the retina-choroid-sclera cascade involves more than one signaling pathway in mammals.

The resultant discrepancies between mRNA and protein levels may be due to posttranscriptional regulatory. Although the overall pattern of protein expression was similar to that of mRNA expression, Gygi et al. [36] found that the correlation between mRNA and protein levels was insufficient to predict protein expression levels from quantitative mRNA data. The incongruent expression between mRNAs and proteins emphasizes the importance of posttranscriptional regulatory mechanisms that can be unveiled only through integrated analyses of both proteins and mRNAs [37]. Translation of individual mRNA species into their encoded proteins may be regulated [38] (e.g., the increase of polyubiquitination leads to accelerated protein degradation, or increased exocytosis leads to the reduced protein level).

In conclusion, the present study found that MT3 can inhibit FDM. TGF-*β*2 and HAS2 may participate in the formation of myopia in the guinea pig. Furthermore, TGF-*β*2 and HAS2 expressions in the retina and choroid were modulated by MT3 in the myopic eyes in guinea pig. This was a preliminary investigation about the expression of TGF-*β*2 and HAS2 in the retina and choroid in FDM. However, the exact signal(s) and cause(s) of changes in TGF-*β*2 and HAS2 expression in the retina and choroid, as well as the manner in which these factors reach the sclera in the development of myopia, remain to be explored in the future.

## Figures and Tables

**Table 1:** Sequences of used primer pairs and length of the amplified sequences.

Primer name	Sequence (5′-3′)	Length (bp)
TGF-*β*2	F: CAAGAGGGATCTTGGCTGGA	190
	R: GGTGAGGGGCTCTAAATCCTG	
HAS2	F: GGAATCACCGCTGCTTACAT	300
	R: TGAGTTCCCATCAATGACCA	
GADPH	F: AAAGGCATCTTGGGCTACACCG	177
	R: ATGAGGTCCACCACCCTGTTG	
